# 6-Nicotinamido-2-naphthoic acid

**DOI:** 10.1107/S1600536812024051

**Published:** 2012-06-02

**Authors:** Yun-Sung Song, Soon W. Lee

**Affiliations:** a Department of Chemistry (BK21), Sungkyunkwan University, Natural Science Campus, Suwon 440-746, Republic of Korea

## Abstract

In the title mol­ecule, C_17_H_12_N_2_O_3_, the naphthalene ring system and the pyridin-3-yl rings are nearly coplanar with a dihedral angle between them of 2.28 (8)°. In the crystal, the hy­droxy and amide N atoms participate in hydrogen bonds, which connect the mol­ecules into a two-dimensional network parallel to (101).

## Related literature

For coordination polymers based on linking ligands with O- and N-donors see: Robin & Fromm, 2006 ▶. For *d*–*f* coordination polymers based on linking ligands with pyrid­yl–carboxyl­ate terminals see: Hu *et al.* (2012 ▶); Chen *et al.* (2010 ▶); Tang *et al.* (2010 ▶); Yue *et al.* (2011 ▶); Zhu *et al.* (2010 ▶). For related potential linking ligands see: Han & Lee, 2012 ▶; Zheng & Lee, 2012 ▶.
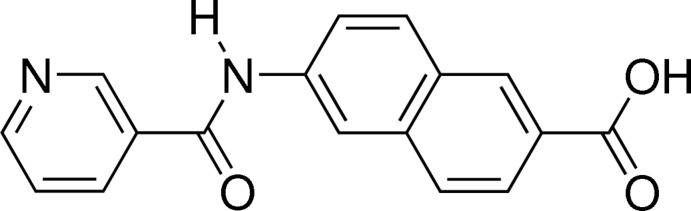


## Experimental

### 

#### Crystal data

C_17_H_12_N_2_O_3_
*M* *_r_* = 292.29
Monoclinic, 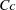

*a* = 25.901 (3) Å
*b* = 6.2097 (7) Å
*c* = 8.6080 (9) Å
β = 103.258 (9)°
*V* = 1347.6 (3) Å^3^
*Z* = 4
Mo *K*α radiation
μ = 0.10 mm^−1^
*T* = 296 K
0.40 × 0.20 × 0.08 mm

#### Data collection

Bruker APEXII CCD diffractometer
Absorption correction: multi-scan (*SADABS*; Sheldrick, 1996 ▶) *T*_min_ = 0.961, *T*_max_ = 0.992
11725 measured reflections
1693 independent reflections
2845 reflections with *I* > 2σ(*I*)
*R*_int_ = 0.035

#### Refinement

*R*[*F* ^2^ > 2σ(*F* ^2^)] = 0.034
*wR*(*F* ^2^) = 0.092
*S* = 1.05
1693 reflections
207 parameters
2 restraints
H atoms treated by a mixture of independent and constrained refinement
Δρ_max_ = 0.24 e Å^−3^
Δρ_min_ = −0.18 e Å^−3^

### 

Data collection: *APEX2* (Bruker (2008 ▶); cell refinement: *SAINT* (Bruker (2008 ▶); data reduction: *SAINT*; program(s) used to solve structure: *SHELXS97* (Sheldrick, 2008 ▶); program(s) used to refine structure: *SHELXL97* (Sheldrick, 2008 ▶); molecular graphics: *SHELXTL* (Sheldrick, 2008 ▶); software used to prepare material for publication: *SHELXTL*.

## Supplementary Material

Crystal structure: contains datablock(s) I, global. DOI: 10.1107/S1600536812024051/mw2071sup1.cif

Structure factors: contains datablock(s) I. DOI: 10.1107/S1600536812024051/mw2071Isup2.hkl

Supplementary material file. DOI: 10.1107/S1600536812024051/mw2071Isup3.cml

Additional supplementary materials:  crystallographic information; 3D view; checkCIF report

## Figures and Tables

**Table 1:** Hydrogen-bond geometry (Å, °)

*D*—H⋯*A*	*D*—H	H⋯*A*	*D*⋯*A*	*D*—H⋯*A*
N2—H2*N*⋯O1^i^	0.92 (3)	2.01 (3)	2.926 (2)	170 (2)
O2—H2*O*⋯N1^ii^	0.84 (3)	1.88 (4)	2.708 (2)	170 (3)

Symmetry codes: (i) 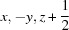; (ii) 
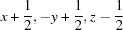
.

**Fig. 1.**
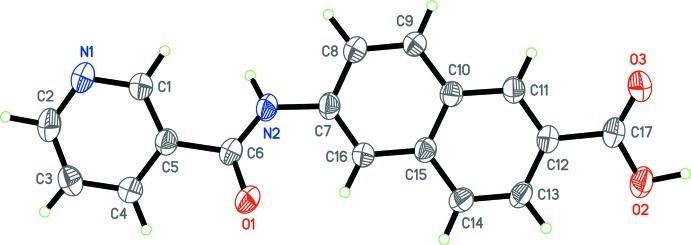
The molecular structure of the title compound showing the atomic numbering and 50% probability displacement ellipsoids.

**Fig. 2.**
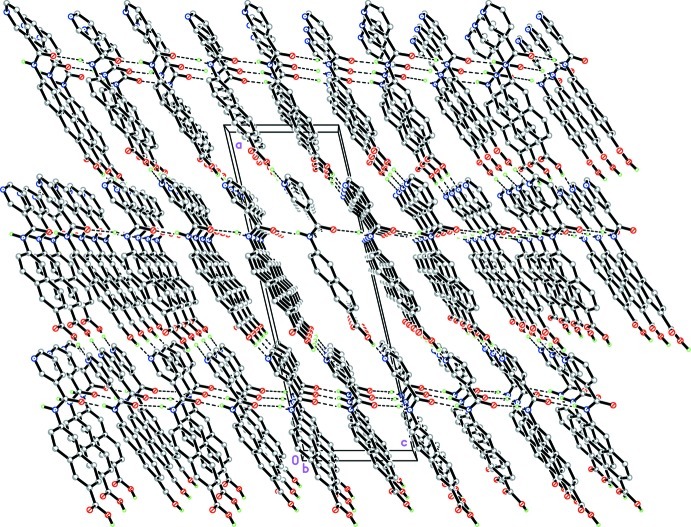
A portion of the crystal packing showing a 2-D H-bonded (dashed lines) network.

## supplementary crystallographic information

### Comment

Bis(pyridyl)- and dicarboxylate-type linking ligands have been typically employed for the preparation of coordination polymers (Robin & Fromm, 2006). The vast majority of known coordination polymers contain either a *d*- or an *f*-block metal. However, several research groups recently prepared polymers containing both *d*- and *f*-block metals within their frameworks by utilizing linking ligands possessing pyridyl–carboxylate terminal groups (Hu *et al.*, 2012; Chen *et al.*, 2010; Tang *et al.*, 2010; Yue *et al.*, 2011; Zhu *et al.*, 2010). Consistent with the hard–soft acid–base concept, the harder oxygen atoms are bonded to the *f*-block metals and the softer nitrogen atoms are bonded to the *d*-block metals in these polymers. Our research group recently reported the structures of two potential linking ligands with pyridyl–carboxylate terminal groups (Han & Lee, 2012; Zheng & Lee, 2012) and here we report the structure of third.

The molecular structure of the title molecule with the atom-labeling scheme is given in Figure 1. The naphthalene and 3-pyridyl rings are nearly coplanar with a dihedral angle between them of 2.28 (8)°. The N2–C6 bond length (1.343 (2) Å) indicates a C–N single bond. The intermolecular O–H···N and N–H···O (carbonyl) hydrogen bonds (Table 1) connect the molecules along the *a*- and *c*-axes, respectively, leading to a 2-D network in the [101] direction (Figure 2).

### Experimental

A stirred mixture of 6-amino-2-naphthoic acid (0.94 g, 5 mmol) and *N*,*N*-dimethyl-4-aminopyridine (0.02 g, 0.17 mmol) in dimethylacetamide (15 mL) was heated at 80 °C for 30 min under argon. The solution was cooled to 10 °C, and nicotinoyl chloride hydrochloride (0.89 g, 5 mmol) was added. The temperature was then raised slowly to 50 °C and was maintained there for 8 h. On addition of dichloromethane to the resulting mixture, a precipitate was formed, which was filtered off and dried under vacuum at 100°C. The product was recrystallized from methanol to give crystals of the title compound (1.22 g, 4.2 mmol, 83.9% yield). mp: 593–595 K (decomp). ^1^H NMR (500 MHz, DMSO-d_6_, d) 11.06 (s, 1H, carboxylic acid OH), 9.35 (s, 1H, amide NH), 8.93 (d, 1H, pyridine proton), 8.71 (d, 1H, pyridine proton), 8.56 (s, 2H, naphthalene proton), 8.14 (d, 1H, pyridine proton), 7.96–7.94 (m, 4H, naphthalene proton), 7.88 (t, 1H, pyridine proton). ^13^C{^1^H} NMR (125 MHz, DMSO-d_6_, d) 167.3, 163.1, 148.7, 145.7, 139.5, 138.4, 135.4, 131.6, 130.2, 129.9, 129.1, 127.8, 127.0, 125.75, 125.0, 121.3, 116.2. IR (KBr, cm^-1^): 3623 (w), 3328 (w), 2925 (*s*), 2640 (*s*), 2372 (*s*), 2075 (*s*), 1800 (*m*), 1621 (*m*), 1551 (*m*), 1291 (*m*), 1195 (*m*), 1018 (*m*), 773 (*m*), 724 (*m*), 678 (*m*), 633 (*m*), 494 (*s*).

### Refinement

All non-hydrogen atoms were refined anisotropically. C-bound H atoms were positioned geometrically [C–H = 0.93–0.97 A] and allowed to ride on their parent atoms, with *U*_iso_(H) = 1.2*U*_eq_(C). The hydrogen atoms attached to N and O were located in a difference Fourier map and refined isotropically.

### Crystal data

**Table:**

C_17_H_12_N_2_O_3_	*F*(000) = 608
*M**_r_* = 292.29	*D*_x_ = 1.441 Mg m^−^^3^
Monoclinic, *C**c*	Mo *K*α radiation, λ = 0.71073 Å
Hall symbol: C -2yc	Cell parameters from 6523 reflections
*a* = 25.901 (3) Å	θ = 3.2–28.5°
*b* = 6.2097 (7) Å	µ = 0.10 mm^−^^1^
*c* = 8.6080 (9) Å	*T* = 296 K
β = 103.258 (9)°	Plate, yellow
*V* = 1347.6 (3) Å^3^	0.40 × 0.20 × 0.08 mm
*Z* = 4	

### Data collection

**Table:**

Bruker APEXII CCD diffractometer	1693 independent reflections
Radiation source: sealed tube	2845 reflections with *I* > 2σ(*I*)
Graphite monochromator	*R*_int_ = 0.035
φ and ω scans	θ_max_ = 28.5°, θ_min_ = 1.6°
Absorption correction: multi-scan (*SADABS*; Sheldrick, 1996)	*h* = −34→34
*T*_min_ = 0.961, *T*_max_ = 0.992	*k* = −8→8
11725 measured reflections	*l* = −11→11

### Refinement

**Table:**

Refinement on *F*^2^	Secondary atom site location: difference Fourier map
Least-squares matrix: full	Hydrogen site location: inferred from neighbouring sites
*R*[*F*^2^ > 2σ(*F*^2^)] = 0.034	H atoms treated by a mixture of independent and constrained refinement
*wR*(*F*^2^) = 0.092	*w* = 1/[σ^2^(*F*_o_^2^) + (0.0641*P*)^2^ + 0.0998*P*] where *P* = (*F*_o_^2^ + 2*F*_c_^2^)/3
*S* = 1.05	(Δ/σ)_max_ < 0.001
1693 reflections	Δρ_max_ = 0.24 e Å^−^^3^
207 parameters	Δρ_min_ = −0.18 e Å^−^^3^
2 restraints	Absolute structure: The absolute structure could not be determined with certainty
Primary atom site location: structure-invariant direct methods	

### Special details

**Table:**

Geometry. All esds (except the esd in the dihedral angle between two l.s. planes) are estimated using the full covariance matrix. The cell esds are taken into account individually in the estimation of esds in distances, angles and torsion angles; correlations between esds in cell parameters are only used when they are defined by crystal symmetry. An approximate (isotropic) treatment of cell esds is used for estimating esds involving l.s. planes.
Refinement. Refinement of *F*^2^ against ALL reflections. The weighted *R*-factor *wR* and goodness of fit *S* are based on *F*^2^, conventional *R*-factors *R* are based on *F*, with *F* set to zero for negative *F*^2^. The threshold expression of *F*^2^ > σ(*F*^2^) is used only for calculating *R*-factors(gt) *etc*. and is not relevant to the choice of reflections for refinement. *R*-factors based on *F*^2^ are statistically about twice as large as those based on *F*, and *R*- factors based on ALL data will be even larger.

### Fractional atomic coordinates and isotropic or equivalent isotropic displacement parameters (Å^2^)

**Table:**

	*x*	*y*	*z*	*U*_iso_*/*U*_eq_	
O1	0.31793 (6)	−0.2140 (2)	0.26571 (17)	0.0391 (3)	
O2	0.62483 (6)	0.3924 (3)	0.1855 (2)	0.0454 (4)	
H2O	0.6456 (12)	0.485 (6)	0.163 (4)	0.059 (8)*	
O3	0.59863 (7)	0.6936 (3)	0.2841 (3)	0.0583 (5)	
N1	0.19038 (7)	−0.1634 (3)	0.5762 (2)	0.0406 (4)	
N2	0.33612 (6)	0.0227 (3)	0.47225 (19)	0.0354 (4)	
H2N	0.3278 (9)	0.070 (4)	0.565 (3)	0.037 (6)*	
C1	0.23346 (8)	−0.0931 (3)	0.5307 (2)	0.0359 (4)	
H1	0.2453	0.0463	0.5580	0.043*	
C2	0.17334 (8)	−0.3618 (4)	0.5349 (3)	0.0432 (5)	
H2	0.1435	−0.4116	0.5666	0.052*	
C3	0.19787 (9)	−0.4971 (4)	0.4473 (3)	0.0430 (5)	
H3	0.1846	−0.6346	0.4200	0.052*	
C4	0.24257 (8)	−0.4253 (4)	0.4005 (2)	0.0386 (4)	
H4	0.2598	−0.5133	0.3409	0.046*	
C5	0.26131 (7)	−0.2192 (3)	0.4442 (2)	0.0301 (4)	
C6	0.30789 (7)	−0.1368 (3)	0.3864 (2)	0.0301 (4)	
C7	0.38118 (7)	0.1288 (3)	0.4401 (2)	0.0307 (4)	
C8	0.39174 (8)	0.3389 (3)	0.5049 (2)	0.0340 (4)	
H8	0.3691	0.4013	0.5616	0.041*	
C9	0.43521 (7)	0.4492 (3)	0.4839 (2)	0.0320 (4)	
H9	0.4420	0.5865	0.5270	0.038*	
C10	0.47019 (7)	0.3577 (3)	0.3975 (2)	0.0283 (4)	
C11	0.51502 (7)	0.4691 (3)	0.3710 (2)	0.0308 (4)	
H11	0.5221	0.6080	0.4106	0.037*	
C12	0.54815 (7)	0.3749 (3)	0.2878 (2)	0.0311 (4)	
C13	0.53849 (8)	0.1624 (3)	0.2294 (2)	0.0350 (4)	
H13	0.5618	0.0978	0.1757	0.042*	
C14	0.49520 (7)	0.0515 (3)	0.2513 (2)	0.0338 (4)	
H14	0.4890	−0.0876	0.2113	0.041*	
C15	0.45962 (7)	0.1469 (3)	0.3345 (2)	0.0285 (4)	
C16	0.41444 (7)	0.0333 (3)	0.3574 (2)	0.0317 (4)	
H16	0.4074	−0.1050	0.3166	0.038*	
C17	0.59290 (7)	0.5052 (4)	0.2540 (2)	0.0356 (4)	

### Atomic displacement parameters (Å^2^)

**Table:**

	*U*^11^	*U*^22^	*U*^33^	*U*^12^	*U*^13^	*U*^23^
O1	0.0371 (7)	0.0478 (8)	0.0382 (6)	−0.0070 (6)	0.0207 (5)	−0.0074 (6)
O2	0.0352 (8)	0.0457 (9)	0.0632 (9)	−0.0036 (7)	0.0274 (7)	0.0042 (7)
O3	0.0498 (10)	0.0452 (10)	0.0917 (13)	−0.0172 (8)	0.0405 (9)	−0.0099 (8)
N1	0.0283 (8)	0.0505 (10)	0.0483 (9)	−0.0018 (7)	0.0197 (7)	−0.0032 (8)
N2	0.0312 (8)	0.0458 (10)	0.0353 (8)	−0.0097 (7)	0.0200 (6)	−0.0055 (7)
C1	0.0283 (9)	0.0382 (10)	0.0452 (10)	−0.0026 (8)	0.0166 (8)	−0.0032 (8)
C2	0.0282 (9)	0.0566 (14)	0.0485 (11)	−0.0062 (9)	0.0166 (8)	0.0062 (10)
C3	0.0375 (11)	0.0382 (11)	0.0551 (12)	−0.0114 (8)	0.0142 (9)	−0.0017 (9)
C4	0.0340 (10)	0.0398 (11)	0.0450 (10)	−0.0033 (7)	0.0156 (8)	−0.0053 (8)
C5	0.0232 (8)	0.0363 (10)	0.0334 (8)	−0.0031 (7)	0.0120 (6)	0.0016 (7)
C6	0.0252 (8)	0.0366 (9)	0.0318 (8)	−0.0011 (7)	0.0132 (6)	0.0023 (7)
C7	0.0255 (9)	0.0385 (11)	0.0308 (8)	−0.0060 (7)	0.0123 (6)	0.0013 (7)
C8	0.0332 (9)	0.0383 (10)	0.0348 (9)	−0.0001 (8)	0.0169 (7)	−0.0021 (7)
C9	0.0334 (10)	0.0317 (9)	0.0343 (8)	−0.0025 (7)	0.0149 (7)	−0.0030 (7)
C10	0.0267 (8)	0.0318 (9)	0.0285 (7)	−0.0003 (7)	0.0109 (6)	0.0021 (6)
C11	0.0281 (9)	0.0324 (9)	0.0337 (8)	−0.0057 (7)	0.0109 (7)	0.0004 (7)
C12	0.0244 (8)	0.0351 (10)	0.0357 (9)	−0.0022 (7)	0.0110 (7)	0.0052 (7)
C13	0.0295 (9)	0.0368 (10)	0.0429 (10)	0.0014 (7)	0.0171 (8)	0.0008 (8)
C14	0.0318 (10)	0.0324 (9)	0.0414 (10)	0.0003 (7)	0.0171 (8)	−0.0022 (7)
C15	0.0266 (9)	0.0331 (9)	0.0289 (7)	−0.0031 (7)	0.0124 (6)	0.0009 (6)
C16	0.0296 (9)	0.0336 (9)	0.0346 (9)	−0.0057 (7)	0.0130 (7)	−0.0013 (7)
C17	0.0256 (9)	0.0445 (12)	0.0392 (9)	−0.0064 (8)	0.0126 (7)	0.0031 (8)

### Geometric parameters (Å, º)

**Table:**

O1—C6	1.225 (2)	C7—C16	1.372 (3)
O2—C17	1.321 (2)	C7—C8	1.421 (3)
O2—H2O	0.84 (3)	C8—C9	1.365 (3)
O3—C17	1.200 (3)	C8—H8	0.9300
N1—C2	1.330 (3)	C9—C10	1.416 (2)
N1—C1	1.338 (2)	C9—H9	0.9300
N2—C6	1.347 (3)	C10—C11	1.414 (2)
N2—C7	1.422 (2)	C10—C15	1.420 (2)
N2—H2N	0.92 (3)	C11—C12	1.369 (3)
C1—C5	1.391 (2)	C11—H11	0.9300
C1—H1	0.9300	C12—C13	1.413 (3)
C2—C3	1.378 (3)	C12—C17	1.496 (2)
C2—H2	0.9300	C13—C14	1.365 (3)
C3—C4	1.384 (3)	C13—H13	0.9300
C3—H3	0.9300	C14—C15	1.420 (2)
C4—C5	1.389 (3)	C14—H14	0.9300
C4—H4	0.9300	C15—C16	1.418 (2)
C5—C6	1.497 (2)	C16—H16	0.9300
			
C17—O2—H2O	104 (2)	C7—C8—H8	120.0
C2—N1—C1	118.18 (17)	C8—C9—C10	120.97 (17)
C6—N2—C7	126.92 (15)	C8—C9—H9	119.5
C6—N2—H2N	120.2 (15)	C10—C9—H9	119.5
C7—N2—H2N	112.9 (15)	C11—C10—C9	122.43 (17)
N1—C1—C5	122.86 (18)	C11—C10—C15	118.87 (15)
N1—C1—H1	118.6	C9—C10—C15	118.69 (15)
C5—C1—H1	118.6	C12—C11—C10	120.79 (18)
N1—C2—C3	123.04 (18)	C12—C11—H11	119.6
N1—C2—H2	118.5	C10—C11—H11	119.6
C3—C2—H2	118.5	C11—C12—C13	120.25 (17)
C2—C3—C4	119.0 (2)	C11—C12—C17	118.50 (18)
C2—C3—H3	120.5	C13—C12—C17	121.19 (17)
C4—C3—H3	120.5	C14—C13—C12	120.39 (17)
C3—C4—C5	118.79 (18)	C14—C13—H13	119.8
C3—C4—H4	120.6	C12—C13—H13	119.8
C5—C4—H4	120.6	C13—C14—C15	120.52 (17)
C4—C5—C1	118.16 (16)	C13—C14—H14	119.7
C4—C5—C6	118.87 (16)	C15—C14—H14	119.7
C1—C5—C6	122.81 (16)	C16—C15—C10	119.90 (15)
O1—C6—N2	123.99 (16)	C16—C15—C14	120.97 (17)
O1—C6—C5	119.50 (17)	C10—C15—C14	119.13 (15)
N2—C6—C5	116.51 (15)	C7—C16—C15	119.58 (18)
C16—C7—C8	120.89 (16)	C7—C16—H16	120.2
C16—C7—N2	122.82 (17)	C15—C16—H16	120.2
C8—C7—N2	116.24 (16)	O3—C17—O2	123.64 (17)
C9—C8—C7	119.96 (16)	O3—C17—C12	123.26 (18)
C9—C8—H8	120.0	O2—C17—C12	113.08 (18)
			
C2—N1—C1—C5	−0.8 (3)	C9—C10—C11—C12	179.58 (16)
C1—N1—C2—C3	−0.2 (3)	C15—C10—C11—C12	−0.8 (3)
N1—C2—C3—C4	0.5 (3)	C10—C11—C12—C13	−1.2 (3)
C2—C3—C4—C5	0.3 (3)	C10—C11—C12—C17	176.03 (16)
C3—C4—C5—C1	−1.2 (3)	C11—C12—C13—C14	2.0 (3)
C3—C4—C5—C6	−176.77 (19)	C17—C12—C13—C14	−175.16 (19)
N1—C1—C5—C4	1.6 (3)	C12—C13—C14—C15	−0.7 (3)
N1—C1—C5—C6	176.89 (18)	C11—C10—C15—C16	−179.01 (19)
C7—N2—C6—O1	0.2 (3)	C9—C10—C15—C16	0.6 (2)
C7—N2—C6—C5	−178.98 (17)	C11—C10—C15—C14	2.0 (2)
C4—C5—C6—O1	23.5 (3)	C9—C10—C15—C14	−178.39 (19)
C1—C5—C6—O1	−151.76 (19)	C13—C14—C15—C16	179.77 (17)
C4—C5—C6—N2	−157.19 (18)	C13—C14—C15—C10	−1.2 (3)
C1—C5—C6—N2	27.5 (3)	C8—C7—C16—C15	−0.8 (3)
C6—N2—C7—C16	−26.4 (3)	N2—C7—C16—C15	−177.94 (16)
C6—N2—C7—C8	156.33 (19)	C10—C15—C16—C7	0.2 (3)
C16—C7—C8—C9	0.6 (3)	C14—C15—C16—C7	179.18 (17)
N2—C7—C8—C9	177.93 (17)	C11—C12—C17—O3	−6.6 (3)
C7—C8—C9—C10	0.2 (3)	C13—C12—C17—O3	170.6 (2)
C8—C9—C10—C11	178.80 (17)	C11—C12—C17—O2	174.43 (16)
C8—C9—C10—C15	−0.8 (3)	C13—C12—C17—O2	−8.4 (3)

### Hydrogen-bond geometry (Å, º)

**Table:**

*D*—H···*A*	*D*—H	H···*A*	*D*···*A*	*D*—H···*A*
N2—H2*N*···O1^i^	0.92 (3)	2.01 (3)	2.926 (2)	170 (2)
O2—H2*O*···N1^ii^	0.84 (3)	1.88 (4)	2.708 (2)	170 (3)

Symmetry codes: (i) *x*, −*y*, *z*+1/2; (ii) *x*+1/2, −*y*+1/2, *z*−1/2.
